# 4-[(*E*)-(4-Chloro­benzyl­idene)amino]-3-methyl-1*H*-1,2,4-triazole-5(4*H*)-thione

**DOI:** 10.1107/S1600536813033527

**Published:** 2013-12-14

**Authors:** B. K. Sarojini, P. S. Manjula, Manpreet Kaur, Brian J. Anderson, Jerry P. Jasinski

**Affiliations:** a Department of Chemistry, P.A. College of Engineering, Nadupadavu 574 153, D.K., Mangalore, India; b Department of Studies in Chemistry, Mangalore University, Mangalagangotri 574 199, Mangalore, India; c Department of Studies in Chemistry, University of Mysore, Manasagangotri, Mysore 570 006, India; d Department of Chemistry, Keene State College, 229 Main Street, Keene, NH 03435-2001, USA

## Abstract

In the title compound, C_10_H_9_ClN_4_S, the dihedral angle between the mean planes of the phenyl and 1*H*-1,2,4-triazole-5(4*H*)-thione rings is 25.3 (9)°. The latter ring is essentially planar, with maximum deviations of 0.010 and −0.010 Å for the ring N atom in the 4-position and ring C atom bearing the methyl group, respectively. An intra­molecular C—H⋯S contact occurs. In the crystal, pairs of weak N—H⋯S inter­actions link the mol­ecules into inversion dimers in the *ac* plane, forming *R*_2_^2^(8) graph-set motifs. In addition, weak π–π inter­actions [centroid–centroid distances = 3.3463 (14) and 3.6127 (13)Å] are observed.

## Related literature   

For the chemistry of Schiff base compounds, see: Dubey & Vaid (1991 ▶); Yadav *et al.* (1994 ▶). For the use of Schiff bases containing different donor atoms in analytical applications and metal coordination, see: Galic *et al.* (2001 ▶); Wyrzykiewicz & Prukah (1998 ▶); Reddy & Lirgappa (1994 ▶). Many compounds containing S and N atoms are anti­hypertensive (Wei *et al.*, 1981 ▶,1982 ▶), analgesic (Thieme *et al.*, 1973*a* ▶,*b* ▶), anti­inflammatory (Dornow *et al.*, 1964 ▶), sedative (Barrera *et al.*,1985 ▶), or fungicidal (Malik *et al.*, 2011 ▶). For the crystal structures of related Schiff bases, see: Jeyaseelan *et al.* (2012 ▶); Devarajegowda *et al.* (2012 ▶); Vinduvahini *et al.* (2011 ▶); Almutairi *et al.* (2012 ▶); Kubicki *et al.* (2012 ▶); Praveen *et al.* (2012 ▶); Kant *et al.* (2012 ▶); Ding *et al.* (2009 ▶). For the crystal structures of Schiff bases reported by our group, see: Sarojini *et al.* (2007*a* ▶,*b* ▶). For standard bond lengths, see: Allen *et al.* (1987 ▶). For π–π stacking, see: Grimme (2008 ▶).
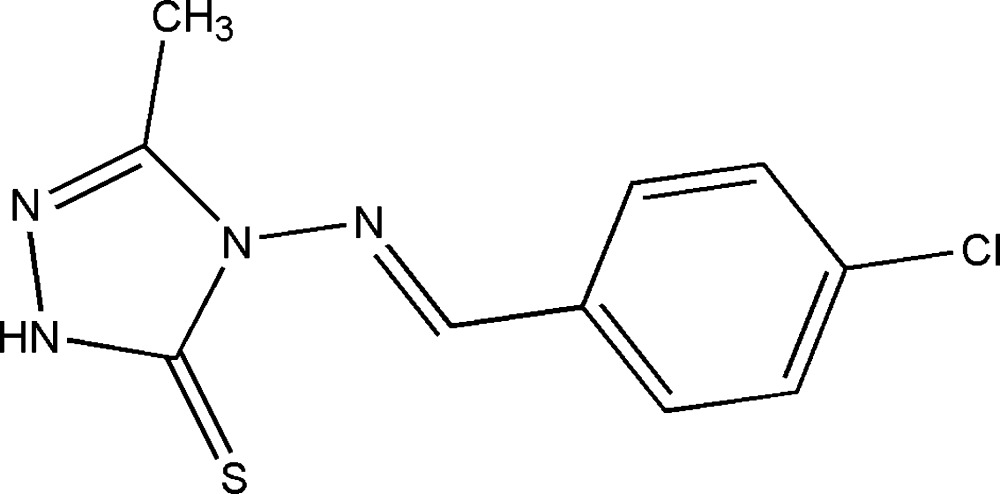


## Experimental   

### 

#### Crystal data  

C_10_H_9_ClN_4_S
*M* *_r_* = 252.72
Triclinic, 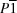

*a* = 7.0718 (8) Å
*b* = 7.2901 (8) Å
*c* = 11.8434 (7) Å
α = 92.778 (7)°
β = 94.986 (7)°
γ = 109.752 (10)°
*V* = 570.50 (10) Å^3^
*Z* = 2
Cu *K*α radiation
μ = 4.49 mm^−1^
*T* = 173 K
0.42 × 0.08 × 0.06 mm

#### Data collection  

Agilent Gemini EOS diffractometer
Absorption correction: multi-scan (*CrysAlis PRO* and *CrysAlis RED*; Agilent, 2012 ▶) *T*_min_ = 0.544, *T*_max_ = 1.000
3413 measured reflections
2195 independent reflections
1860 reflections with *I* > 2σ(*I*)
*R*_int_ = 0.030

#### Refinement  

*R*[*F* ^2^ > 2σ(*F* ^2^)] = 0.043
*wR*(*F* ^2^) = 0.120
*S* = 1.02
2195 reflections
146 parameters
H-atom parameters constrained
Δρ_max_ = 0.34 e Å^−3^
Δρ_min_ = −0.39 e Å^−3^

### 

Data collection: *CrysAlis PRO* (Agilent, 2012 ▶); cell refinement: *CrysAlis PRO*; data reduction: *CrysAlis RED* (Agilent, 2012 ▶); program(s) used to solve structure: *SUPERFLIP* (Palatinus & Chapuis, 2007 ▶); program(s) used to refine structure: *SHELXL2012* (Sheldrick, 2008 ▶); molecular graphics: *OLEX2* (Dolomanov *et al.*, 2009 ▶); software used to prepare material for publication: *OLEX2*.

## Supplementary Material

Crystal structure: contains datablock(s) I. DOI: 10.1107/S1600536813033527/qm2102sup1.cif

Structure factors: contains datablock(s) I. DOI: 10.1107/S1600536813033527/qm2102Isup2.hkl

Supporting information file. DOI: 10.1107/S1600536813033527/qm2102Isup3.cml

Additional supporting information:  crystallographic information; 3D view; checkCIF report

## Figures and Tables

**Table 1:** Hydrogen-bond geometry (Å, °)

*D*—H⋯*A*	*D*—H	H⋯*A*	*D*⋯*A*	*D*—H⋯*A*
N4—H4⋯S1^i^	0.86	2.43	3.2926 (19)	176
C1—H1⋯S1	0.93	2.62	3.199 (2)	121

Symmetry code: (i) 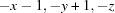.

**Fig. 1.**
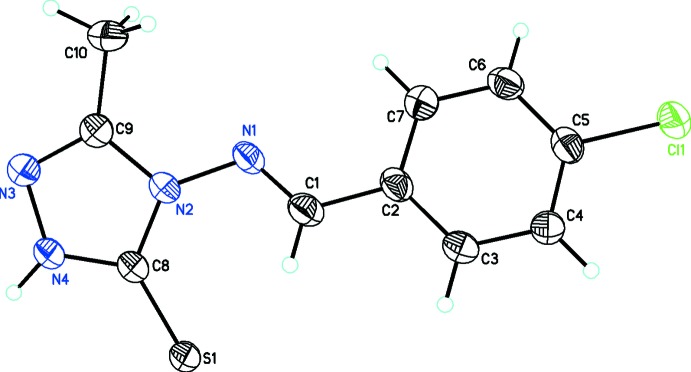
ORTEP drawing of (I) (C10H9ClN4S) showing the labeling scheme with 50% probability displacement ellipsoids.

**Fig. 2.**
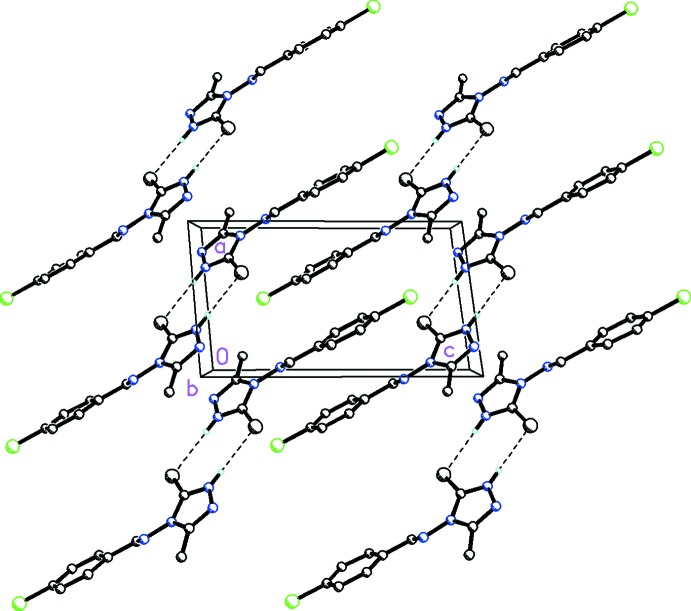
Molecular packing for (I) viewed along the b axis. Dashed lines indicate weak N4—H4···S1 intermolecular interactions forming R22(8) graph set motifs linking the molecules into dimers diagonally in the ac plane. H atoms not involved in hydrogen bonding have been removed for clarity.

**Fig. 3.**
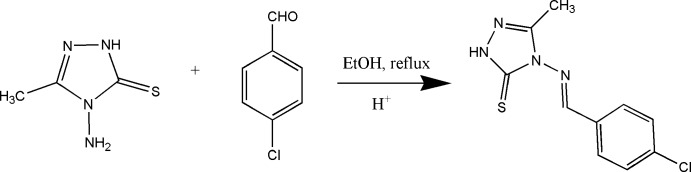
Synthesis scheme for (I).

## supplementary crystallographic information

### 1. Comment

During the last few decades, there has been a considerable interest in the chemistry of Schiff base compounds (Dubey & Vaid, 1991; Yadav *et al.*, 1994). Schiff bases, containing different donor atoms, also find use in analytical applications and metal coordination (Galic *et al.*, 2001; Wyrzykiewicz & Pruka, 1998; Reddy & Lirgappa, 1994). Since many compounds containing sulfur and nitrogen atoms are antihypertensive (Wei *et al.*, 1981,1982), analgesic (Thieme *et al.*, 1973*a*,*b*), antiinflammatory (Dornow *et al.*, 1964), sedative (Barrera *et al.*,1985), or fungicidal (Malik *et al.*, 2011), synthesis of the corresponding heterocyclic compounds could be of interest from the viewpoint of chemical reactivity and biological activity. The crystal structures of some of the related Schiff bases viz: 3-ethyl-4-[(*E*)-(4-fluorobenzylidene)amino]-1*H*-1,2,4-triazole-5(4*H*)-thione (Jeyaseelan *et al.*, 2012); 4-[(*E*)-(4-fluorobenzylidene)amino]-3-methyl-1*H*-1,2,4-triazole-5(4*H*)-thione (Devarajegowda *et al.*, 2012); 3-[2-(2,6-dichloro-anilino)benzyl]-4-[(4-methoxybenzylidene)amino]-1*H*-1,2,4-triazole-5(4*H*)-thione (Vinduvahini *et al.*, 2011); 3-(adamantan-1-yl)-1-[(4-ethylpiperazin-1-yl)methyl]-4-[(*E*)-(4-hydroxy-benzylidene)amino]-1*H*-1,2,4-triazole-5(4*H*)-thione (Almutairi *et al.*, 2012); 4-{(2*E*)-2-[1-(4-Methoxyphenyl)ethylidene]hydrazinyl}-8-(trifluoromethyl)quinoline (Kubicki *et al.*, 2012); (*E*)-*N*'-(4-Methoxybenzylidene)-2-*m*-tolylacetohydrazide (Praveen *et al.*, 2012); (1*Z*)-1-[(2*E*)-3-(4-bromophenyl)-1-(4-fluorophenyl)prop-2-en-1-ylidene]-2-(2,4-dinitrophenyl)hydrazine (Kant *et al.*, 2012); (*E*)-3-(2-ethoxyphenyl)-4-(2-fluorobenzylideneamino)-1*H*-1,2,4-triazole-5(4*H*)-thione (Ding *et al.*,2009) have been reported. Crystal structures of some of the Schiff bases were also reported from our group (Sarojini *et al.*, 2007*a*,*b*). In continuation of our work on the synthesis of acetamides derivatives, we report herein the crystal structure of the title compound, C_10_H_9_ClN_4_S, (I).

In the title compound, (I), C_10_H_9_ClN_4_S, the dihedral angle between the mean planes of the phenyl ring and the1*H*-1,2,4-triazole-5(4*H*)-thione ring is 25.3 (9)° (Fig.1). The 1,2,4-triazole-5(4*H*)-thione ring is essentially planar with a maximum deviation of 0.010Å and -0.010Å for the N2 nitrogen and C9 carbon atom, respectively. Bond lengths and bond angles are in normal ranges (Allen *et al.*, 1987).In the crystal, weak N4—H4···S1 intermolecular interactions forming R_2_^2^(8) graph set motifs link the molecules into dimers diagonally in the ac plane (Fig. 2). In addition, weak Cg–Cg π–π intermolecular interactions are observed which may contribute to crystal packing (Cg1–Cg1 = 3.3463 (14)Å; -x, 1-y, -z; Cg2–Cg2 = 3.6127 (13)Å; 1-x, 2-y, 1-z; Cg1 = N2/C81/N4/N3/C9; Cg2 = C2–C7) (Grimme, 1987). No classical hydrogen bonds were observed.

### 2. Experimental

To a suspension of 4-chlorobenzaldehyde (1.405 g, 0.01 mol) in ethanol (15 ml), 4-amino-5-methyl-2,4-dihydro-3H-1,2,4-triazole- 3-thione (0.01 mol, 1.3 g) was added and heated to get a clear solution. To this a few drops of conc.H_2_SO_4_ was added as a catalyst and refluxed for 36 h on a water bath. The precipitate formed was filtered and recrystallized from methanol to get the titled compound. The single crystals were grown from methanol (M.P.:.463 - 465 K).

### 3. Refinement

All of the H atoms were placed in their calculated positions and then refined using the riding model with Atom—H lengths of 0.93Å (CH), 0.96Å (CH_3_) or 0.86Å (NH). Isotropic displacement parameters for these atoms were set to 1.2 (CH, NH) or 1.5 (CH_3_) times *U*_eq_ of the parent atom.Idealised Me refined as rotating groups.

### Crystal data

**Table:**

C_10_H_9_ClN_4_S	*Z* = 2
*M**_r_* = 252.72	*F*(000) = 260
Triclinic, *P*1	*D*_x_ = 1.471 Mg m^−^^3^
*a* = 7.0718 (8) Å	Cu *K*α radiation, λ = 1.54184 Å
*b* = 7.2901 (8) Å	Cell parameters from 1400 reflections
*c* = 11.8434 (7) Å	θ = 3.8–72.2°
α = 92.778 (7)°	µ = 4.49 mm^−^^1^
β = 94.986 (7)°	*T* = 173 K
γ = 109.752 (10)°	Irregular, colourless
*V* = 570.50 (10) Å^3^	0.42 × 0.08 × 0.06 mm

### Data collection

**Table:**

Agilent Gemini EOS diffractometer	2195 independent reflections
Radiation source: Enhance (Cu) X-ray Source	1860 reflections with *I* > 2σ(*I*)
Detector resolution: 16.0416 pixels mm^-1^	*R*_int_ = 0.030
ω scans	θ_max_ = 72.3°, θ_min_ = 3.8°
Absorption correction: multi-scan (*CrysAlis PRO* and *CrysAlis RED*; Agilent, 2012)	*h* = −8→8
*T*_min_ = 0.544, *T*_max_ = 1.000	*k* = −8→8
3413 measured reflections	*l* = −10→14

### Refinement

**Table:**

Refinement on *F*^2^	Primary atom site location: structure-invariant direct methods
Least-squares matrix: full	Hydrogen site location: inferred from neighbouring sites
*R*[*F*^2^ > 2σ(*F*^2^)] = 0.043	H-atom parameters constrained
*wR*(*F*^2^) = 0.120	*w* = 1/[σ^2^(*F*_o_^2^) + (0.0694*P*)^2^] where *P* = (*F*_o_^2^ + 2*F*_c_^2^)/3
*S* = 1.02	(Δ/σ)_max_ < 0.001
2195 reflections	Δρ_max_ = 0.34 e Å^−^^3^
146 parameters	Δρ_min_ = −0.39 e Å^−^^3^
0 restraints	

### Special details

**Table:**

Geometry. All esds (except the esd in the dihedral angle between two l.s. planes) are estimated using the full covariance matrix. The cell esds are taken into account individually in the estimation of esds in distances, angles and torsion angles; correlations between esds in cell parameters are only used when they are defined by crystal symmetry. An approximate (isotropic) treatment of cell esds is used for estimating esds involving l.s. planes.

### Fractional atomic coordinates and isotropic or equivalent isotropic displacement parameters (Å^2^)

**Table:**

	*x*	*y*	*z*	*U*_iso_*/*U*_eq_	
Cl1	0.50541 (9)	0.96033 (9)	0.77738 (5)	0.03752 (19)	
S1	−0.33106 (9)	0.64930 (9)	0.16551 (5)	0.03564 (19)	
N1	0.0401 (3)	0.4905 (3)	0.27296 (16)	0.0295 (4)	
N2	−0.0729 (3)	0.4344 (3)	0.16664 (15)	0.0287 (4)	
N3	−0.1813 (3)	0.2442 (3)	0.00660 (17)	0.0353 (4)	
N4	−0.2891 (3)	0.3680 (3)	0.02140 (16)	0.0317 (4)	
H4	−0.3862	0.3696	−0.0271	0.038*	
C1	0.0775 (3)	0.6667 (3)	0.31206 (19)	0.0311 (5)	
H1	0.0364	0.7520	0.2684	0.037*	
C2	0.1852 (3)	0.7342 (3)	0.42579 (19)	0.0289 (5)	
C3	0.2005 (3)	0.9179 (3)	0.4726 (2)	0.0320 (5)	
H3	0.1460	0.9957	0.4301	0.038*	
C4	0.2955 (3)	0.9876 (3)	0.5816 (2)	0.0318 (5)	
H4A	0.3026	1.1095	0.6131	0.038*	
C5	0.3788 (3)	0.8714 (3)	0.64183 (19)	0.0307 (5)	
C6	0.3682 (3)	0.6889 (3)	0.5977 (2)	0.0329 (5)	
H6	0.4265	0.6136	0.6400	0.039*	
C7	0.2703 (3)	0.6195 (3)	0.4903 (2)	0.0333 (5)	
H7	0.2606	0.4959	0.4605	0.040*	
C8	−0.2301 (3)	0.4851 (3)	0.11732 (19)	0.0291 (5)	
C9	−0.0524 (3)	0.2861 (3)	0.0975 (2)	0.0322 (5)	
C10	0.1023 (4)	0.1949 (4)	0.1262 (2)	0.0431 (6)	
H10A	0.0590	0.1060	0.1837	0.065*	
H10B	0.2284	0.2949	0.1541	0.065*	
H10C	0.1195	0.1248	0.0594	0.065*	

### Atomic displacement parameters (Å^2^)

**Table:**

	*U*^11^	*U*^22^	*U*^33^	*U*^12^	*U*^13^	*U*^23^
Cl1	0.0398 (3)	0.0455 (3)	0.0282 (3)	0.0191 (3)	−0.0056 (2)	−0.0031 (2)
S1	0.0364 (3)	0.0421 (3)	0.0329 (3)	0.0242 (3)	−0.0099 (2)	−0.0080 (2)
N1	0.0255 (9)	0.0372 (10)	0.0263 (10)	0.0134 (7)	−0.0047 (7)	0.0010 (7)
N2	0.0270 (9)	0.0321 (9)	0.0274 (10)	0.0132 (7)	−0.0046 (7)	−0.0007 (7)
N3	0.0369 (10)	0.0397 (10)	0.0325 (11)	0.0212 (8)	−0.0063 (8)	−0.0057 (8)
N4	0.0318 (10)	0.0394 (10)	0.0272 (10)	0.0198 (8)	−0.0066 (8)	−0.0030 (8)
C1	0.0260 (10)	0.0337 (11)	0.0334 (12)	0.0113 (8)	−0.0029 (9)	0.0044 (9)
C2	0.0208 (10)	0.0344 (11)	0.0296 (12)	0.0087 (8)	−0.0020 (8)	0.0007 (9)
C3	0.0264 (10)	0.0364 (12)	0.0355 (12)	0.0153 (9)	−0.0017 (9)	0.0010 (9)
C4	0.0282 (10)	0.0353 (11)	0.0339 (12)	0.0152 (9)	−0.0004 (9)	−0.0041 (9)
C5	0.0248 (10)	0.0406 (12)	0.0260 (11)	0.0112 (9)	0.0007 (8)	−0.0001 (9)
C6	0.0331 (11)	0.0350 (11)	0.0328 (12)	0.0155 (9)	−0.0015 (9)	0.0064 (9)
C7	0.0346 (11)	0.0307 (11)	0.0342 (12)	0.0122 (9)	−0.0009 (10)	−0.0001 (9)
C8	0.0284 (10)	0.0330 (11)	0.0269 (11)	0.0138 (8)	−0.0036 (8)	0.0003 (9)
C9	0.0314 (11)	0.0350 (11)	0.0311 (12)	0.0148 (9)	−0.0021 (9)	−0.0024 (9)
C10	0.0420 (14)	0.0460 (14)	0.0481 (16)	0.0288 (12)	−0.0086 (12)	−0.0060 (12)

### Geometric parameters (Å, º)

**Table:**

Cl1—C5	1.748 (2)	C2—C7	1.402 (3)
S1—C8	1.689 (2)	C3—H3	0.9300
N1—N2	1.395 (2)	C3—C4	1.390 (3)
N1—C1	1.275 (3)	C4—H4A	0.9300
N2—C8	1.378 (3)	C4—C5	1.377 (3)
N2—C9	1.379 (3)	C5—C6	1.381 (3)
N3—N4	1.378 (2)	C6—H6	0.9300
N3—C9	1.301 (3)	C6—C7	1.379 (3)
N4—H4	0.8600	C7—H7	0.9300
N4—C8	1.333 (3)	C9—C10	1.484 (3)
C1—H1	0.9300	C10—H10A	0.9600
C1—C2	1.463 (3)	C10—H10B	0.9600
C2—C3	1.390 (3)	C10—H10C	0.9600
			
C1—N1—N2	116.28 (19)	C4—C5—C6	122.1 (2)
C8—N2—N1	131.27 (18)	C6—C5—Cl1	119.20 (18)
C8—N2—C9	108.25 (18)	C5—C6—H6	120.4
C9—N2—N1	119.99 (18)	C7—C6—C5	119.2 (2)
C9—N3—N4	103.61 (18)	C7—C6—H6	120.4
N3—N4—H4	122.9	C2—C7—H7	119.8
C8—N4—N3	114.29 (18)	C6—C7—C2	120.4 (2)
C8—N4—H4	122.9	C6—C7—H7	119.8
N1—C1—H1	120.1	N2—C8—S1	129.63 (17)
N1—C1—C2	119.8 (2)	N4—C8—S1	127.48 (17)
C2—C1—H1	120.1	N4—C8—N2	102.87 (18)
C3—C2—C1	118.6 (2)	N2—C9—C10	122.4 (2)
C3—C2—C7	118.7 (2)	N3—C9—N2	110.94 (19)
C7—C2—C1	122.6 (2)	N3—C9—C10	126.6 (2)
C2—C3—H3	119.3	C9—C10—H10A	109.5
C4—C3—C2	121.3 (2)	C9—C10—H10B	109.5
C4—C3—H3	119.3	C9—C10—H10C	109.5
C3—C4—H4A	120.9	H10A—C10—H10B	109.5
C5—C4—C3	118.2 (2)	H10A—C10—H10C	109.5
C5—C4—H4A	120.9	H10B—C10—H10C	109.5
C4—C5—Cl1	118.70 (18)		
			
Cl1—C5—C6—C7	−179.28 (17)	C1—C2—C3—C4	178.0 (2)
N1—N2—C8—S1	5.3 (4)	C1—C2—C7—C6	−179.2 (2)
N1—N2—C8—N4	−173.2 (2)	C2—C3—C4—C5	1.4 (3)
N1—N2—C9—N3	174.80 (19)	C3—C2—C7—C6	−0.4 (3)
N1—N2—C9—C10	−6.5 (3)	C3—C4—C5—Cl1	178.09 (17)
N1—C1—C2—C3	−171.5 (2)	C3—C4—C5—C6	−0.8 (3)
N1—C1—C2—C7	7.2 (3)	C4—C5—C6—C7	−0.4 (4)
N2—N1—C1—C2	176.53 (18)	C5—C6—C7—C2	1.0 (3)
N3—N4—C8—S1	−177.92 (17)	C7—C2—C3—C4	−0.8 (3)
N3—N4—C8—N2	0.6 (3)	C8—N2—C9—N3	2.0 (3)
N4—N3—C9—N2	−1.5 (3)	C8—N2—C9—C10	−179.3 (2)
N4—N3—C9—C10	179.8 (2)	C9—N2—C8—S1	177.00 (18)
C1—N1—N2—C8	−35.9 (3)	C9—N2—C8—N4	−1.5 (2)
C1—N1—N2—C9	153.2 (2)	C9—N3—N4—C8	0.5 (3)

### Hydrogen-bond geometry (Å, º)

**Table:**

*D*—H···*A*	*D*—H	H···*A*	*D*···*A*	*D*—H···*A*
N4—H4···S1^i^	0.86	2.43	3.2926 (19)	176
C1—H1···S1	0.93	2.62	3.199 (2)	121

Symmetry code: (i) −*x*−1, −*y*+1, −*z*.
